# Effects of landscape features on population genetic variation of a tropical stream fish, Stone lapping minnow, *Garra cambodgiensis*, in the upper Nan River drainage basin, northern Thailand

**DOI:** 10.7717/peerj.4487

**Published:** 2018-03-07

**Authors:** Chaowalee Jaisuk, Wansuk Senanan

**Affiliations:** 1 Department of Aquatic Science, Faculty of Science, Burapha University, Chon Buri, Thailand; 2 Department of Animal Science and Fisheries, Faculty of Science and Agricultural Technology, Rajamangala University of Technology Lanna Nan Campus, Nan, Thailand

**Keywords:** Microsatellite variation, Spatial genetic variation, Landscape genetics, Tropical stream fish, *Garra cambodgiensis*, Upper Nan River

## Abstract

Spatial genetic variation of river-dwelling freshwater fishes is typically affected by the historical and contemporary river landscape as well as life-history traits. Tropical river and stream landscapes have endured extended geological change, shaping the existing pattern of genetic diversity, but were not directly affected by glaciation. Thus, spatial genetic variation of tropical fish populations should look very different from the pattern observed in temperate fish populations. These data are becoming important for designing appropriate management and conservation plans, as these aquatic systems are undergoing intense development and exploitation. This study evaluated the effects of landscape features on population genetic diversity of *Garra cambodgiensis,* a stream cyprinid*,* in eight tributary streams in the upper Nan River drainage basin (*n* = 30–100 individuals/location), Nan Province, Thailand. These populations are under intense fishing pressure from local communities. Based on 11 microsatellite loci, we detected moderate genetic diversity within eight population samples (average number of alleles per locus = 10.99 ± 3.00; allelic richness = 10.12 ± 2.44). Allelic richness within samples and stream order of the sampling location were negatively correlated (*P* < 0.05). We did not detect recent bottleneck events in these populations, but we did detect genetic divergence among populations (Global *F*_ST_ = 0.022, *P* < 0.01). The Bayesian clustering algorithms (TESS and STRUCTURE) suggested that four to five genetic clusters roughly coincide with sub-basins: (1) headwater streams/main stem of the Nan River, (2) a middle tributary, (3) a southeastern tributary and (4) a southwestern tributary. We observed positive correlation between geographic distance and linearized *F*_ST_ (*P* < 0.05), and the genetic differentiation pattern can be moderately explained by the contemporary stream network (STREAMTREE analysis, *R*^2^ = 0.75). The MEMGENE analysis suggested genetic division between northern (genetic clusters 1 and 2) and southern (clusters 3 and 4) sub-basins. We observed a high degree of genetic admixture in each location, highlighting the importance of natural flooding patterns and possible genetic impacts of supplementary stocking. Insights obtained from this research advance our knowledge of the complexity of a tropical stream system, and guide current conservation and restoration efforts for this species in Thailand.

## Introduction

The historical and contemporary river and stream landscapes have a profound effect on the population demographic and genetic processes of resident fish species (Castric, Bonny & Bernatchez, 2001; Beneteau, Mandrak & Heath, 2009; Hopken, Douglas & Douglas, 2012; Davis, Wieman & Berendzen, 2015; Crookes & Shaw, 2016). A tropical river landscape is the outcome of a long geomorphological process, and was less affected by the latest glaciation period at the end of the Pleistocene era (approximately 100,000 to 10,000 years ago) than rivers in temperate regions. Southeast Asia is a part of the Indo-Malaysian zoogeographical subregion (Proches & Ramdhani, 2012), with a long biogeographical history for its aquatic fauna, and is a hotspot for their biodiversity. Wallace (1876) divided Southeast Asia into the Indochinese, Sundaic and Philippine subregions. Thailand, a part of the Indochinese subregion, housed a major river valley (called Siam) connecting the Tibetan Plateau and the Sunda shelf (Woodruff, 2010). The ‘Siam’ system evolved into the current Chao Phraya River system within the last three million years.

Genetic variation in natural populations reflects population history and the evolutionary potential of a species (Frankham, Ballou & Briscore, 2010), an important consideration for aquatic conservation (Allendorf & Luikart, 2007). The presence of population subdivision within a fish species is the result of an interplay between restricted gene flow and independent genetic changes within isolated populations (Hedrick, 2011). In the absence of gene flow, conspecific populations generally will diverge from one another as a result of genetic drift, natural selection and mutations (Freeland, 2005). In addition, fish dispersal strategies and life-history traits determine the magnitude of landscape effects in shaping patterns of genetic variation (Pilger et al., 2017).

Although we recognize a general pattern of landscape effects on population genetic diversity, the boundaries for genetic divergence specific to a river system also vary, depending upon the scale and complexity of local landscapes, fish life history and fish population dynamics. Genetic differentiation often is detected at a drainage basin level (Neville, Dunham & Peacock, 2006; Beneteau, Mandrak & Heath, 2009) or upstream and downstream within a drainage (Barson, Cable & Van Oosterhout, 2009). Geographic factors encouraging population division include geographic distance between locations (Lamphere & Blum, 2012; Crookes & Shaw, 2016; Beneteau, Mandrak & Heath, 2009; Hopken, Douglas & Douglas, 2012), the presence of barriers (Neville, Dunham & Peacock, 2006; Yamamoto et al., 2004), the complexity of a stream network (Pilger et al., 2017) and habitat fragmentation (Sterling et al., 2012). Models often used to describe genetic population structure of stream fishes include the stream hierarchy model (Meffe & Vrijenhoek, 1988) and isolation-by-distance (Bohonak, 2002). The magnitude of population divergence depends also on life-history traits such as body size (Hughes et al., 2012; Pilger et al., 2017), habitat preference (Lamphere & Blum, 2012), and migratory behavior within the life cycle (Barson, Cable & Van Oosterhout, 2009). Most of our current understanding of landscape genetics has been derived from temperate or sub-tropical species and may not provide adequate guidance for conservation and management of fish populations in a tropical setting, as in Southeast Asia. Therefore, to design locally relevant management strategies, data generated from fish populations from a tropical system will be necessary.

The Nan River is the largest tributary (740 km) of the Chao Phraya River. Similarly to other tropical rivers, the Nan River is under the influence of seasonal monsoons. The entire Nan River drainage basin covers 34,682.04 km^2^ and is divided by the large Sirikit Dam and its reservoir into upper and lower portions. The upper Nan River drainage basin captures all highland headwater streams draining into the main stem of the Nan River and occupies approximately 1/3 of the entire Nan River basin. Due to its heterogeneous landscape containing some pristine highland headwater streams, the upper Nan River basin harbors high fish species diversity—at least 108 species (Lothongkham, 2008) of the 600 species found in Thailand. Similar to other tropical aquatic systems, rapid habitat alterations, including deforestation, and heavy fishing pressure are threats to the sustainability of fish populations.

The Stone lapping minnow or False Siamese algae eater (*Garra cambodgiensis*) is a small-bodied (4–10 cm) cyprinid and a habitat specialist inhabiting rocky bottoms of fast- flowing sections of small-sized and medium-sized streams. This species is widely distributed in large river basins in Southeast Asia, including those of the Chao Phraya, Mekong and Meklong rivers (Lothongkham, 2008) as well as in southern China, India and the Middle East to northern and central Africa (Kullander & Fang, 2004). This species occurs in almost all tributaries of the Nan River. This species typically reaches maturation at 2–3 years old. Life-history characteristics related to dispersal ability of this species have not been well documented. However, local fishermen often harvest them during the spawning season (May-August) when breeders congregate in flooded lowland areas adjacent to the stream/river. Females are especially valuable. As the water recedes, the breeders disperse, as well as their fry. It is likely that their semi-buoyant eggs and fry drift via stream flow throughout the stream network.

This study aimed to evaluate the effects of landscape features on population genetic structure of *G. cambodgiensis* in the upper Nan River drainage basin based on polymorphic co-dominant microsatellite DNA markers. This species is currently heavily exploited and, as a small-bodied fish, it may be greatly affected by landscape features. Lack of knowledge of population genetics of these populations has led to haphazard attempts to supplement headwater streams with hatchery-produced fry. A better understanding of the landscape genetics of *G. cambodgiensis* will serve as a foundation for genetic monitoring and provide guidance for conservation and restoration of wild populations of this tropical stream fish.

## Materials and Methods

### Study system and field sampling

**Figure 1:**
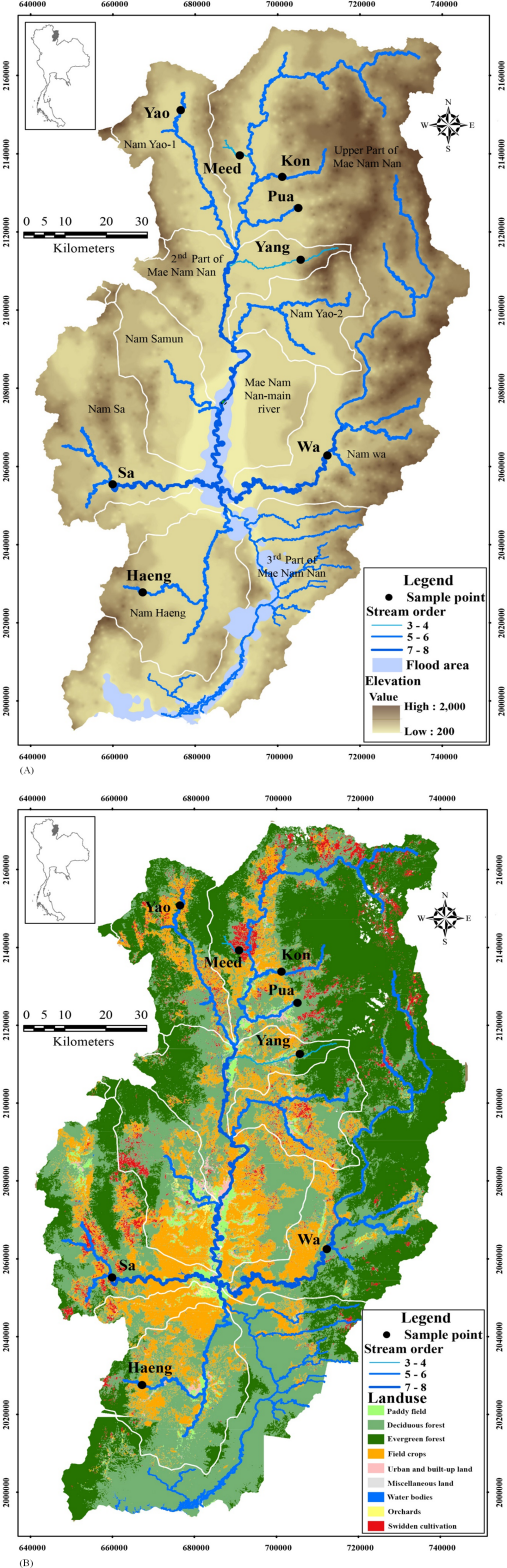
Locations of population samples of *Garra cambodgiensis* in the upper Nan River drainage basin, Thailand taken from November–December 2016. The map also illustrates (A) stream orders, flooded areas and elevation and (B) land use types in the drainage basin. GIS data provided by the Nan Provincial Administrative Organization.

The upper Nan River basin is located in Nan Province, in the northern region of Thailand. It lies between 1990471 and 217000 N and 640000 and 750000 E. The catchment covers approximately 15,564.12 km^2^ (Fig. 1). The landscape of the upper Nan River watershed is heterogeneous, including both flat and mountainous terrain. Eighty-five percent of the land area of Nan Province is forested mountains and highlands. Major land uses in this watershed include forest (deciduous and evergreen forest), agriculture (field crops and swidden cultivation, an agricultural system entailing temporary clearings of forested plots for few crop growing cycles, after which the plots are abandoned and allowed to revert to their natural vegetation; during the fallow period, the farmer usually moves to another plot) and paddy fields (Table 1, Fig. 1). The drainage basin contains ten sub-basins, namely Upper part of Mae Nam Nan, Nam Yao-1, Second part of Mae Nam Nan, Nam Yao-2, Nam Samun, Third part of Mae Nam Nan, Nam Sa, Nam Wa, Nam Haeng and Fourth part of Mae Nam Nan (in which Sirikit Dam is located). Eight of the ten sub-basins of the upper Nan River contain the species of interest, *G. cambodgiensis*.

We collected a total of 397 *G. cambodgiensis* adult individuals from six sub-basins (30–100 fin clips per site) during the dry season, November–December 2016. Fish distribution was not affected by flooding. Individuals were sampled from their feeding habitats (Table 1, Fig. 1). Fin clips were preserved in 95% ethanol for further analysis. The sampling sites represented watersheds of various sizes, land-use types and stream orders. These characteristics were used to test the effects of landscape attributes on local genetic variation and population divergence.

**Table 1:** Landscape characteristics of *Garra cambodgiensis* sampling locations in the upper Nan River drainage basin, Thailand.

Location (Sampling code)	Geographic co-ordinates (UTM)		Sub basin^a^	Sub-basin area (km^2^)	Elevation (MSL)	Major land use types (%) (within 4 km radius of the sampling location) Forest/Agriculture/ Paddy field	Stream order at sampling locations	Distance to the main channel of Nan River (km)	Sample size (*N*)
	*X*	*Y*							
Meed River (Meed)	690744	2138971	Upper part of Mae Nam Nan	2,222.34	500	45.19/52.58/0.78	4	4.60	46
Kon River (Kon)	701057	2133505			300	63.37/21.58/8.93	5	8.97	46
Pua River (Pua)	704968	2125543			292	28.57/31.89/26.76	4	17.89	46
Yao River (Yao)	676400	2150534	Nam Yao-1	787.73	329	26.24/73.34/0	5	58.96	46
Yang River (Yang)	705554	2112381	Second part of Mae Nam Nan	2,200.39	365	52.87/35.36/7.38	3	22.99	100
Sa River (Sa)	659914	2055068	Nam Sa	778.40	347	49.07/49.96/0.44	7	45.68	41
Haeng River (Haeng)	667162	2027495	Nam Haeng	1,043.80	404	37.29/54.69/3.92	5	65.64	42
Wa River (Wa)	712063	2062370	Nam Wa	3,375.80	252	61.88/34.99/0.52	7	64.00	30

**Notes.**

a Pakoksung & Koontanakulvong (2015).

The Institute of Animals for Scientific Purposes Development (IAD) endorsed a license for animal use for the lead investigator (license number U 1 05299 2559). The Research Ethics Committee of the Faculty of Science, Burapha University, approved the research in June 2016 (Research Ethics Committee meeting report 3/2559).

### DNA extraction and microsatellite genotyping

Genomic DNA was extracted using a salt extraction protocol modified from Aljanabi & Martinez (1997). We amplified 11 microsatellite loci previously identified for *Garra* spp. using polymerase chain reactions (PCR). These loci were GC203, GC187 and Sa197 (Jaisuk et al., 2014), Gar3, Gar6, Gar8, Gar9, Gar13 (Su et al., 2013), PH8A, JQSO and HOLN (Kirchner et al., 2014). A forward primer for each primer pair was fluorescently labeled at the 5′ end (FAM, HEX, VIC or ROX). The total volume of a polymerase chain reaction was 10 ul, consisting of 10 ng of DNA template, 0.1 mM of each primer in a primer pair, and 5 ul of *iTaq* mastermix solution (iNtRON BIOTECHNOLOGY, Gyeonggi-do, South Korea). The PCRs were performed in a thermal cycler (BioRad, MJ Mini Cycler, Milan, Italy) with the following temperature profile: a cycle of 94 °C for two minutes; 40 cycles of denaturation at 94 °C for 30 s, an annealing temperature specific to each primer pair for 30 s (48 °C for PH8A and Gar8; 54 °C for Gar9 and JQSO; 58 °C for GC203, GC187, Gar3, Gar6, Gar13 and 60 °C for Sa197 and HOLN), and elongation at 72 °C for 30 s; and a final elongation at 72 °C for 5 min. The PCR products were submitted to a commercial genetic analysis service (First BASE Laboratories Sdn Bhd, Selangor, Malaysia) for electrophoresis and genotyping on an ABI3730XL DNA analyser. Scores were determined relative to an internal size standard (LIZ 500) using the GeneMapper software v.3.0 (Applied Biosystems, Foster City, CA, USA).

### Genetic diversity within populations

We estimated the following standard genetic diversity indices: average number of alleles (*A*), effective number of alleles (*A*_*e*_), heterozygosity (observed, *H*_*o*_ and expected, *H*_*e*_ heterozygosities), and inbreeding coefficient (*F*_is_) using GenAlEx v.6.5 (Peakall & Smouse, 2006). Unequal sample sizes among population samples can lead to incomparable allelic diversity across samples. To account for unequal sample sizes among population samples, we estimated allelic richness (*A*_*r*_) based on the rarefaction approach implemented in *F* stat v.2.9.3, (Goudet, 2001) (smallest sample size across all samples = 30). To test for statistically significant differences in genetic diversity measures among population samples, we used Mann–Whitney *U*-tests (Crookes & Shaw, 2016).

We tested for deviation of observed genotypes from those expected under the Hardy-Weinberg equilibrium by the Markov chain Monte Carlo method of exact probability test implemented in the software Genepop v.4.0 (Rousset, 2008). *P*-values were estimated from 10,000 dememorization numbers, in 100 batches with 5,000 iterations per batch. For the statistical inference, the *P*-value was adjusted using Bonferroni correction for multiple tests (Rice, 1989). Genotyping errors due to non-amplified alleles (null alleles), short allele dominance (large allele dropout) and the scoring of stutter peaks were assessed based on the Chakraborty (Chakraborty, Srinivasan & Daiger, 1993) and Dempster (Dempster, Laird & Rubin, 1977) methods implemented in the programs MICRO-CHECKER v.2.2.3 (Van Oosterhout et al., 2004) and FreeNA (Chapuis & Estoup, 2007), respectively. Moreover, to account for the effects of null alleles on the detection of population genetic structure, the FreeNA program estimated pairwise *F*_ST_ values (Weir, 1996) based on allele frequencies corrected for null alleles (i.e., ENA, excluding null alleles, Chapuis & Estoup, 2007).

### Estimation of *N*_*e*_ and the presence of recent bottlenecks

The contemporary effective population size (*N*_*e*_) of each sample/genetic cluster was calculated based on two methods, the linkage disequilibrium (LD) method (Do et al., 2014) and sibship method (Wang, 2016), implemented in NeEstimator v.2 and COLONY v.2.05.1, respectively. For the LD method, the lowest allele frequency used was 0.01 and putative 95% confidence intervals were calculated by a parametric method (Do et al., 2014). For the sib-ship approach, COLONY uses maximum likelihood to estimate probabilities of full and half siblings of a sample of individuals taken from a population of interest.

To detect evidence of a recent bottleneck, we evaluated the rapid increase in heterozygosities compared to those expected across loci under a mutation-drift equilibrium assuming a two-phase model of microsatellite evolution (TPM), which is most appropriate for empirical microsatellite data (Di Rienzo et al., 1994; Piry, Luikart & Cornuet, 1999), with 90% single-step mutations and 10% multiple-step mutations and 1,000 replications in BOTTLENECK v.1.2.02 (Piry, Luikart & Cornuet, 1999). The program used Wilcoxon’s test to infer the significance of the expected heterozygosity (*H*_*e*_) excess compared to the values expected under the mutation-drift equilibrium (*H*_eq_).

### Genetic differentiation among populations

We employed both conventional and model-based approaches to assess genetic divergence among population samples and to assess the potential admixture within a sample. Analysis of molecular variance (AMOVA; Excoffier, Laval & Schneider, 2005), an analogue to ANOVA, allowed for the partitioning of overall variance into variation among-sub-basin, among-sites within sub-basins, and among-individuals. We then estimated pairwise *F*_ST_ values using an AMOVA framework with an exact test *P*-value for a given *F*_ST_ based on random permutation procedures (1,000 permutations) using the software ARLEQUIN v.3.5 (Excoffier & Lischer, 2010). The level of significance was adjusted for multiple simultaneous tests using the sequential Bonferroni procedure (Rice, 1989).

Cluster analysis was performed based upon the Nei’s genetic distance (Nei, 1978) matrix, and a dendrogram was constructed using the unweighted pair group method with arithmetic averaging (UPGMA) algorithm using the Poppr R package (Kamvar, Tabima & Grunwald, 2014). We constructed a consensus dendrogram showing bootstrap support values for nodes (percentages based on 1000 bootstrap replicates).

To further explore spatial genetic variation, we used two Bayesian clustering models implemented in the software STRUCTURE v.2.3.4 (Pritchard, Stephens & Donnelly, 2000; Hubisz et al., 2009) and TESS v.2.3 (Chen et al., 2007; Francois & Durand, 2010). The two models differ in their utility of spatial information, with TESS incorporating geographic coordinates of individuals in the analysis. Both approaches analyzed multilocus genotypes of individuals to determine a likely number of genetic clusters (*K*) and estimated membership coefficients (for a given *K* value) for each individual. For STRUCTURE analysis, the most likely *K* value for the dataset was determined by a method proposed by (Evanno, Regnaut & Goudet, 2005) based on the difference in log probability of data between successive *K* values (i.e., Δ*K* statistics). A *K* value with the highest rate of change would be the probable *K* value for the data set. To obtain these probability values, we simulated a range of *K* values between 1 and 9, with 20 replicated runs for each value of *K* and a burn-in period of 25,000 and 100,000 Markov chain Monte Carlo (MCMC) iterations (these parameter settings were recommended by the software developer). We used the admixture model with correlated allele frequencies, and default parameter settings. This model is typically a starting point for most analyses. It is a reasonably flexible model for dealing with many of the complexities of real populations (Pritchard, Wen & Falush, 2010). The Δ*K* statistic plot was generated by STRUCTURE HARVESTER v.0.6.94 (Earl & vonHoldt, 2012).

For TESS analysis, the most likely *K* value was selected based on the rapid decline of Deviance Information Criterion (*DIC*) values averaged over 20 simulations between subsequent *K* values. To obtain the *DIC* values, the analysis was performed using the CAR admixture model, which assumes spatial autocorrelation of the genomes of individuals in closer geographical proximity compared with those further apart. The spatial interaction parameter (*ψ*) was set to the default value of 0.6 for analysis. TESS was run with a burn-in of 30,000 sweeps followed by 50,000 sweeps, with 20 independent runs conducted for each value of *K*, from 2 to 9. For each *K*, both STRUCTURE and TESS estimated a membership coefficient, accounting for sampling location, for each individual. These coefficients reflected the genetic admixture level within individuals (if any). The display of membership coefficients of individuals for each *K* value was generated using the Pophelper R web app v.1.0.10 (Francis, 2017). Both programs were useful for exploring genetic differentiation patterns.

To determine possible gene flow among populations, we jointly estimated long-term migration rates among populations and historical effective population size using the MCMC maximum-likelihood method implemented in the software Migrate-n v.3.2.1 (Beerli, 2012). This software estimates theta (Θ), which equals four times the effective population size, *N*_*e*_, times the mutation rate, *μ* (4 *N*_*e*_*μ*), and a migration rate parameter *M*, which is the immigration rate *m* divided by the mutation rate *μ*. Search criteria in Migrate-n were set to 10 short chains of 10,000 steps, 500 trees recorded and three long chains of 100,000 steps, 5,000 trees recorded and a static heating scheme with the following temperatures 1.0, 1.3, 3.0, and 10,000. Microsatellite mutation was modeled as a continuous Brownian and stepwise process. Migrate-n was run six times with parameter values starting from *F*_ST_-based estimates, and the distribution of parameter values was compared across runs to ensure overlap of 95% C.I. Effective sample size was 7000 for all runs. Values of long-term, historical estimates of gene flow (*M*) were converted to proportion of migrants (*m*). The conversion was calculated using the formula: *m* = *M* µ(Apodaca, Rissler & Godwin, 2012) where µ= 5.56 ×10^−4^ (Yue, David & Orban, 2007). Historical *N*_*e*_ values were estimated from the Θ values divided by 4 *μ*.

### Spatial genetic analysis

To assess spatial pattern of genetic variation, we examined correlations between landscape features and genetic diversity (i.e., allelic richness) as well as with genetic differentiation (i.e., *F*_ST_). We tested the effects of sampling site elevation, stream order, distance to the main channel of the Nan River, and percentages of major land-use types within a 4-km radius of sampling locations (i.e., forest, agriculture, and paddy field) on allelic richness using Pearson correlation analysis (IBM SPSS Statistics v.20). We also tested for the isolation by distance (IBD) pattern by performing Mantel’s tests (Mantel, 1967) on linearized *F*_ST_ values (*F*_ST_∕(1 − *F*_ST_) and pair-wise stream distance among sampling locations, calculated by Google Earth (log transformed). The Mantel tests (*P*-values were generated from 1,000 permutations) were performed in the ecodist package in R (Goslee & Urban, 2007). In addition, we determined whether genetic diversity reflected contemporary patterns of stream connectivity by analyzing the model fit between the pairwise *F*_ST_ values and the number of stream sections connecting sampling locations based on the statistical methods in the software STREAMTREE (Kalinowski et al., 2008). This approach evaluates the model fit, indicated by a coefficient of determination (*R*^2^), between two neighbor-joining dendrograms, one from genetic distance data and another from stream network data sections.

To evaluate the extent to which landscape variables contribute to genetic differences among samples (quantified as linearized *F*_ST_), we followed an approach used by Pilger et al. (2017) by using an information theoretic approach in combination with multiple-regression-on-distance matrices (MRM). The best candidate multiple regression models were selected based on Akaike’s information criterion (*AIC*) scores, adjusted for small sample size (*AIC*c), and Akaike weights (*w*_*i*_). These values were calculated using the AICcmodavg package in R (Mazerolle, 2017). Candidate models with the lowest *AIC*c scores (Δ*AIC*c < 2.0) and highest weights (*w*_*i*_ > 0.10) were retained (Burnham & Anderson, 2002) and evaluated for the contribution of explanatory variables to the overall fit of the model (MRM *R*^2^) using the function MRM implemented in the ecodist package in R (Goslee & Urban, 2007). Significance of the respective MRM models was assessed by a permutation test (1000 permutations).

We also performed multivariate analysis based on genotypes and spatial coordinates. We visualized the signal for spatial genetic variation using an approach proposed by Galpern et al. (2014) based on a combination of a multivariate analysis (Moran’s eigenvector maps) of multilocus genotypes and a regression between a genetic distance matrix (i.e., proportion of shared alleles) and landscape predictors. The analyses were implemented in the MEMGENE package in R language (Galpern et al., 2014). The results from the MEMGENE analysis (i.e., scores of MEMGENE variables of sampling coordinates) were superimposed on the upper Nan River map.

## Results

### Microsatellite variability and genetic diversity within *G. cambodgiensis* populations from the Nan River, Thailand

All microsatellite loci were polymorphic in all populations sampled (Table 2). The average number of alleles per locus across samples ranged from 6.25 ± 1.56 (Gar8) to 14.63 ± 4.27 (Gar6). The average effective number of alleles per locus ranged from 3.02 ± 0.83 (Gar9) to 8.35 ± 1.28 (HOLN), and the average allelic richness ranged from 5.62 ± 0.99 (Gar8) to 12.72 ± 2.70 (Gar6). The expected heterozygosities averaged across samples ranged from 0.64 ± 0.10 (Gar9) to 0.88 ± 0.02 (HOLN).

**Table 2:** Average allelic variability (mean ±  SD) at 11 microsatellite loci of *Garra cambodgiensis* populations in the upper Nan River, Thailand. The indices included the sample size (*N*), number of alleles per locus (*A*), effective number of alleles (*A*_*e*_), allelic richness (*A*_*r*_), observed heterozygosity (*H*_*o*_), expected heterozygosity (*H*_*e*_), fixation index (*F*_*is*_) and estimated null allele frequencies.

	*N*	*A*	*A*_*e*_	*A*_*r*_	*H*_*o*_	*H*_*e*_	*F*_is_	Null allele frequency
Locations								
Meed	43.46 ± 1.62	10.91 ± 2.64	6.35 ± 1.90	10.35 ± 2.38	0.51 ± 0.14	0.83 ± 0.06	0.38 ± 0.15	0.18 ± 0.07
Kon	44.00 ± 2.45	11.64 ± 2.93	5.59 ± 1.54	10.61 ± 2.36	0.56 ± 0.15	0.81 ± 0.06	0.31 ± 0.17	0.14 ± 0.07
Pua	45.27 ± 1.14	12.09 ± 1.88	6.24 ± 2.36	11.05 ± 1.87	0.63 ± 0.19	0.79 ± 0.14	0.22 ± 0.15	0.10 ± 0.06
Yao	45.27 ± 1.05	11.36 ± 2.50	5.90 ± 1.61	10.56 ± 2.11	0.65 ± 0.12	0.82 ± 0.05	0.21 ± 0.12	0.10 ± 0.06
Yang	95.64 ± 5.40	13.36 ± 3.52	6.04 ± 2.42	10.86 ± 2.62	0.55 ± 0.15	0.80 ± 0.10	0.31 ± 0.16	0.14 ± 0.07
Sa	39.36 ± 2.06	9.82 ± 1.64	5.58 ± 1.99	9.48 ± 1.59	0.63 ± 0.15	0.79 ± 0.11	0.21 ± 0.14	0.09 ± 0.06
Wa	30.00 ± 0.00	8.00 ± 1.92	4.31 ± 1.70	7.64 ± 1.92^*^	0.62 ± 0.13	0.73 ± 0.11	0.14 ± 0.17	0.07 ± 0.05
Haeng	40.00 ± 2.37	11.09 ± 2.78	6.39 ± 2.41	10.45 ± 2.59	0.54 ± 0.16	0.81 ± 0.11	0.33 ± 0.17	0.15 ± 0.08
All samples	47.88 ± 18.82	10.99 ± 3.00	5.80 ± 2.12	10.12 ± 2.44	0.59 ± 0.16	0.80 ± 0.10	0.26 ± 0.17	0.12 ± 0.07
Each locus								
Gar3	48.38 ± 19.74	10.88 ± 1.69	6.47 ± 1.10	10.07 ± 1.04	0.70 ± 0.05	0.84 ± 0.03	0.17 ± 0.06	0.07 ± 0.03
Gar6	49.00 ± 19.16	14.63 ± 4.27	6.19 ± 1.50	12.72 ± 2.70	0.67 ± 0.09	0.83 ± 0.06	0.19 ± 0.12	0.09 ± 0.05
Gar8	49.63 ± 19.71	6.25 ± 1.56	3.63 ± 0.48	5.62 ± 0.99	0.58 ± 0.08	0.72 ± 0.05	0.24 ± 0.07	0.09 ± 0.05
Gar9	43.88 ± 14.61	10.00 ± 2.50	3.02 ± 0.83	9.02 ± 1.78	0.40 ± 0.11	0.64 ± 0.10	0.38 ± 0.18	0.16 ± 0.07
Gar13	47.00 ± 20.33	10.13 ± 2.20	5.17 ± 1.27	9.35 ± 1.66	0.60 ± 0.05	0.79 ± 0.07	0.24 ± 0.08	0.11 ± 0.03
GC187	48.25 ± 17.58	9.75 ± 1.09	6.38 ± 0.93	9.51 ± 1.03	0.60 ± 0.08	0.84 ± 0.03	0.28 ± 0.11	0.13 ± 0.05
GC203	49.00 ± 19.55	11.75 ± 0.66	7.54 ± 1.24	11.32 ± 0.66	0.72 ± 0.11	0.86 ± 0.02	0.17 ± 0.12	0.09 ± 0.05
HOLN	48.00 ± 18.75	12.75 ± 1.56	8.35 ± 1.28	12.01 ± 1.18	0.60 ± 0.10	0.88 ± 0.02	0.32 ± 0.11	0.15 ± 0.05
JQSO	49.63 ± 19.71	10.75 ± 1.20	7.21 ± 0.95	10.10 ± 0.87	0.77 ± 0.08	0.86 ± 0.02	0.11 ± 0.09	0.05 ± 0.04
Sa197	45.88 ± 18.90	12.38 ± 2.45	6.76 ± 2.18	11.54 ± 2.18	0.39 ± 0.14	0.83 ± 0.07	0.54 ± 0.17	0.24 ± 0.07
PH8A	48.00 ± 18.71	11.63 ± 2.91	3.08 ± 0.80	10.11 ± 2.47	0.45 ± 0.13	0.65 ± 0.11	0.32 ± 0.15	0.14 ± 0.06

**Notes.**

* indicates a statistically significant *P* value for Mann–Whitney *U*-test (*P* < 0.05).

There was no evidence of stutter products or allelic dropout. Nevertheless, MICRO-CHECKER and FreeNA analysis suggested the presence of null alleles at some loci, with the frequencies ranging from 0.05 ± 0.04 (JQSO) to 0.24 ± 0.07 (Sa197). However, for a given locus, we did not observe consistent null allele frequencies across populations. This observation suggested that any deviation from an ideal population may be due to population properties rather than the presence of null alleles. Moreover, the FreeNA analysis suggested only a slight change in pairwise *F*_ST_ values when including or excluding the estimated frequencies of null alleles (Table S1). We therefore retained the original dataset for population divergence analyses.

Based on within-sample genetic diversity measures, the Wa sample had the lowest allelic richness (7.64 ± 1.92^∗^, *P* < 0.05, Mann–Whitney *U*-test), but had comparable values for other measures to the remaining samples. For allelic diversity, the average number of alleles per locus (*A*) ranged from 8.00 ± 1.92 (Wa) to 12.09 ± 1.88 (Pua), the effective number of alleles per locus (*A*_*e*_) ranged from 4.31 ±1.70 (Wa) to 6.39 ± 2.41 (Haeng), and allelic richness (*A*_*r*_) ranged from 7.64 ± 1.92 (Wa) to 11.05 ± 1.87 (Pua). Heterozygosity values were comparable across all samples. Of the 88 sample-locus cases (8 samples ×11 loci), 40 cases showed significant deviation from HWE (*P* < 0.00057, after Bonferroni correction = 0.05/88). All of the deviations were heterozygote deficiencies (*H*_*o*_ <*H*_*e*_) (Table S2), which we attribute to the mixing of partially differentiated populations, as noted below.

### Effective population size and evidence of recent bottlenecks

The two approaches yielded different *N*_*e*_ estimates for a given population, with the linkage disequilibrium method resulting in an estimate with higher variance compared to the sib-ship methods. Based on the linkage disequilibrium method, Pua had the lowest *N*_*e*_ estimate (*N*_*e*_ = 260.4, 95% CI [153.2–761.6]) and other populations sampled had an estimate with a large 95% CI (upper bound = infinity). The sib-ship method resulted in the lowest *N*_*e*_ estimate in Wa (*N*_es_ = 40, 95% CI [25–47]); most populations had comparable estimates. We did not detect significant heterozygote excess in any of the samples under the two-phase mutation model (TPM). Additionally, the mode-shift test showed a normal L-shaped distribution pattern of the allele frequencies in all samples. The results implied the lack of bottleneck events in the recent history of these populations (Table 3).

**Table 3:** Estimates and 95% confidence intervals of contemporary effective population size (*N*_*e*_) and the detection of bottlenecks based on Wilcoxon’s test under the two-phase mutation model (TMP) for eight population samples at 11 microsatellite loci.

	Effective population size						Bottleneck test
	Based on linkage disequilibrium			Based on sib-ship			
	*N*_*e*_	95% Confidence intervals		*N*_es_	95% Confidence intervals		
Sample		Lower bound	Upper bound		Lower bound	Upper bound	TPM (*P*-value)
Meed	408.3	175.9	infinite	64	43	100	0.83
Kon	Infinite	407.1	infinite	63	40	99	0.17
Pua	260.4	153.2	761.6	58	38	95	0.21
Yao	422.1	192.4	infinite	54	36	85	0.46
Yang	1,554.5	556.6	infinite	97	71	137	0.10
Sa	Infinite	273	infinite	44	27	73	0.70
Wa	406.6	101.7	infinite	40	25	47	0.58
Haeng	Infinite	339.6	infinite	70	48	107	0.76

### Genetic differentiation among samples and population genetic structure

*G. cambodgiensis* populations in the upper Nan River were genetically heterogeneous. Both conventional and Bayesian genetic analyses suggested genetic divergence among the eight samples. Global *F*_ST_ estimated by the AMOVA framework was 0.02249 (*P* < 0.01). Genetic variation from among sub-basins, among sites within sub-basins, and within sites contributed 2%, 2%, and 96% of total variation, respectively. The pairwise *F*_ST_ values ranged from 0.003 (Yao and Sa) to 0.053 (Wa and Sa) (Table 4). All samples were genetically different from each other except for two sample pairs, Kon-Meed and Yao-Sa (*P*-value after adjusting for multiple comparisons = 0.05/28).

**Table 4:** Pairwise *F*_ST_ values (below diagonal) and geographic distance (km) (above diagonal) among Garra cambodgiensis population samples in the upper Nan River, Thailand. Significant values of *F*_ST_ are underlined.

	Meed	Kon	Pua	Yao	Yang	Sa	Wa	Haeng
Meed		22.48	49.14	100.52	70.81	181.50	203.18	210.96
Kon	0.003		44.60	95.98	66.27	176.96	198.64	206.42
Pua	0.020	0.013		87.16	57.45	168.14	189.82	197.60
Yao	0.018	0.016	0.017		88.32	199.01	220.69	228.47
Yang	0.019	0.016	0.016	0.022		156.67	178.35	186.13
Sa	0.034	0.029	0.026	0.003	0.029		113.04	120.82
Wa	0.036	0.028	0.026	0.044	0.041	0.053		135.64
Haeng	0.021	0.021	0.020	0.021	0.015	0.029	0.051	

**Figure 2:**
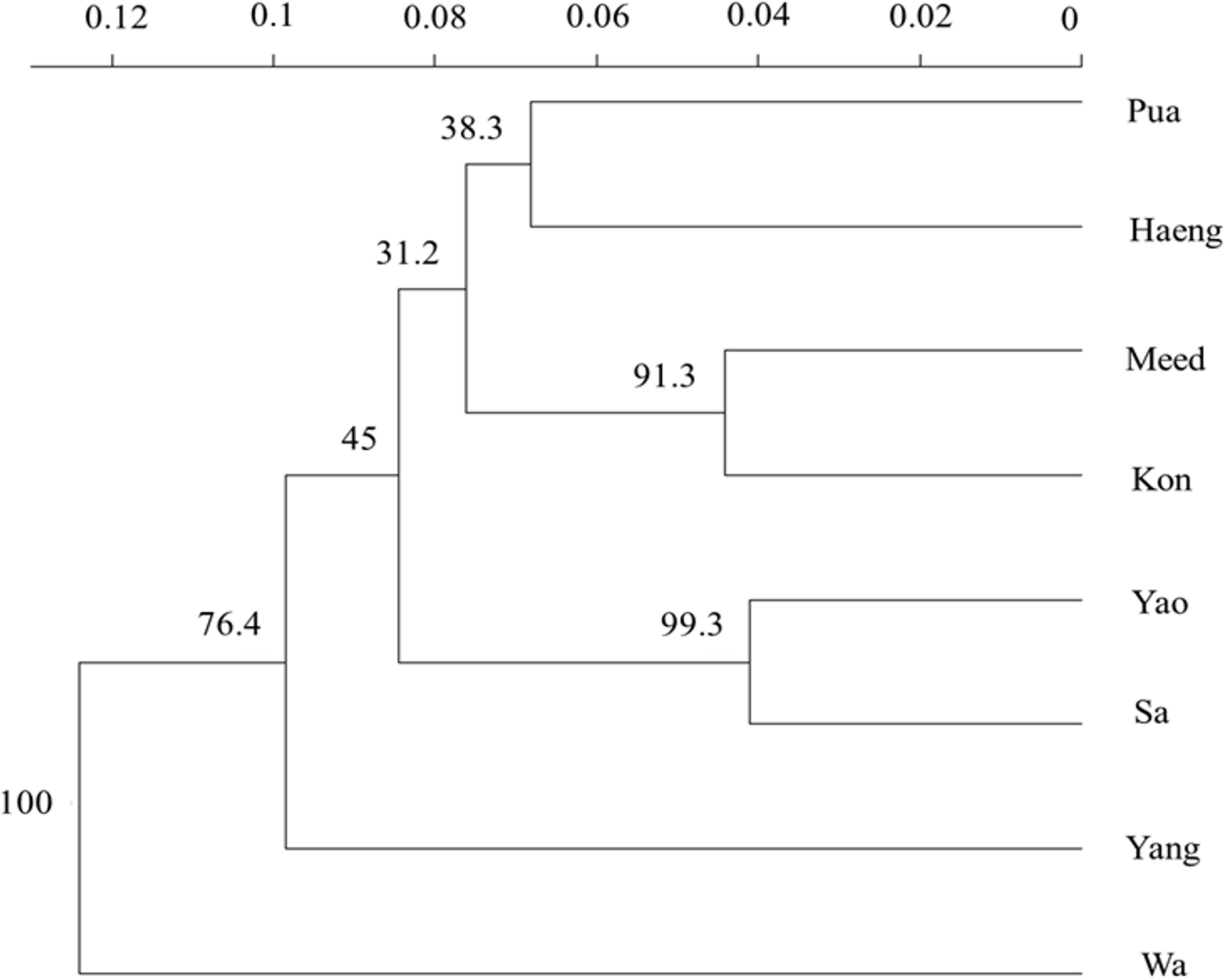
UPGMA dendrogram of eight population samples of *Garra cambodgiensis* based on Nei’s genetic distance (Nei, 1978) (indicated by a scale bar) with 1,000 bootstrap replicates at 11 microsatellite loci (bootstrap values are shown at nodes).

**Figure 3:**
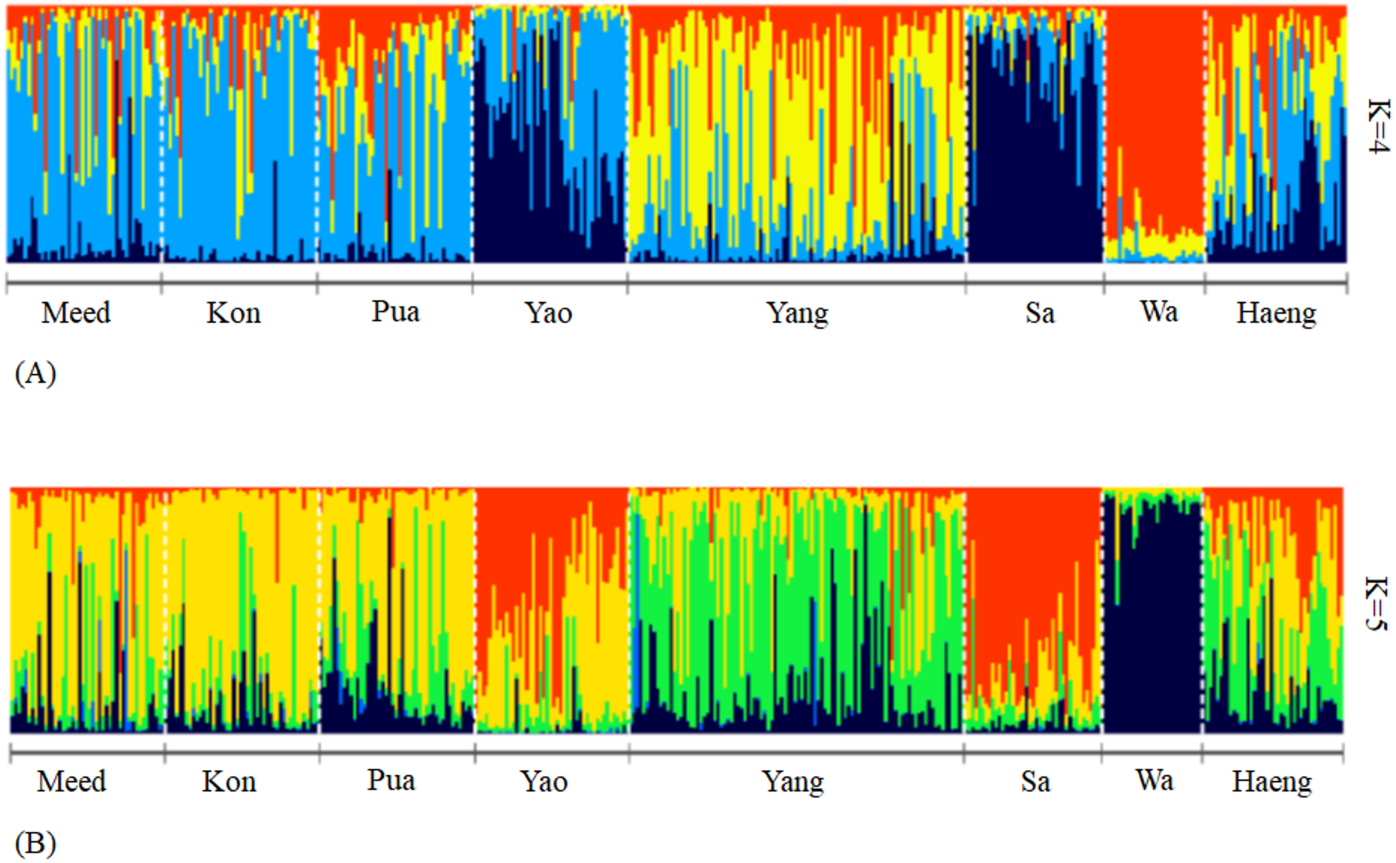
Bar plot of membership coefficients of individuals assigned to genetic clusters (*K* = 4 and 5) generated by a Bayesian clustering algorithm, implemented in the TESS software. The individual coefficients (vertical bars) were grouped by population samples. Membership to each cluster is represented by a different color. The bar plot illustrates (A) four and (B) five genetic clusters.

The UPGMA dendrogram based on Nei’s genetic distance also formed robust clusters of Yao and Sa (bootstrap value = 99.3) and of Kon and Meed (bootstrap value = 91.3) (Fig. 2). The samples from the upstream sites of the Nan River, including Kon, Meed, Yang, and Pua, were in the same cluster. It is interesting to note that the most downstream sample, Haeng, was also included in this cluster. The Bayesian clustering algorithms implemented in STRUCTURE and TESS suggested five and four possible genetic clusters, respectively (Fig. 3). Because TESS provided a clearer picture of population subdivision, we present only the TESS results. The distribution of individual membership coefficients arranged by populations sampled suggested that Wa was genetically distinct from other samples. The bar plots also suggested genetic similarity among the three adjacent sites, Meed, Kon and Pua. Even though a large number of individuals in the Yang sample comprised a separate genetic cluster, some individuals shared a genetic composition with those in Kon, Meed, Pua and Haeng. The Sa and Yao samples had a similar genetic composition, but Yao contained higher numbers of individuals with admixed ancestry compared to Sa (Fig. 3). Moreover, it is apparent that most of the population samples contained admixed individuals with ancestry from more than one genetic cluster. Although there is no location-specific cluster, some spatial patterns were apparent. These divisions included: (1) the headwater tributaries (Meed/Kon/Pua) and main stem of the Nan River (including Haeng), (2) a middle tributary (Yang), (3) an eastern tributary (Wa), and (4) a western tributary (Sa). The most downstream site, Haeng, was comprised of admixed individuals containing similar proportions of all four genetic clusters.

### Migration

Estimates of genetically effective migration suggested some dispersal among the populations, although the migration rates were not substantial (Table 5). The results from Migrate-n showed some gene flow among sub-basins, with the migration rates per generation ranging from 3.7 ×10^−4^ (Wa received from Pua) to 12.5 ×10^−4^ (Yang received from Pua). Migration from Yang to some northern streams (Meed, Kon and Pua) were similar (*m* = 10.2 × 10^−4^–12.3 ×10^−4^). Adjacent rivers also had slightly higher migration rates than those more distantly located. For example, Yang received immigrants from Pua (*m* = 12.5 × 10^−4^) and Wa received immigrants from Sa at higher rates (*m* = 7.7 × 10^−4^) than other, more distant sites. The most downstream site, Haeng, received immigrants from all samples. Most samples showed estimated immigration rates comparable to emigration rates. Numbers of migrants per generation (*N*_*e*_*m*) between sites were low (<0.7). Estimated migration rates suggested that Yang was a likely donor population within the upper Nan River drainage basin, especially to the upstream tributaries, with *N*_*e*_*m* estimates ranging from 0.313 (to Haeng)-0.636 (to Meed) (Table 5).

**Table 5:** Estimated historical gene flow among sampled populations based on variation at 11 microsatellite loci. *M*_*ij*_ is an immigration rate from population *i* to *j*, scaled by mutation rate and *m*_*ij*_ is the immigration rate from *i* to *j*. Numbers in bold are highest migration rate in a receiving population.

	Donor population																
	Meed		Kon		Pua		Yao		Yang		Sa		Wa		Haeng		
	*M*_*ij*_	*m*_*ij*_ (10^−4^)	*M*_*ij*_	*m*_*ij*_ (10^−4^)	*M*_*ij*_	*m*_*ij*_ (10^−4^)	*M*_*ij*_	*m*_*ij*_ (10^−4^)	*M*_*ij*_	*m*_*ij*_ (10^−4^)	*M*_*ij*_	*m*_*ij*_ (10^−4^)	*M*_*ij*_	*m*_*ij*_ (10^−4^)	*M*_*ij*_	*m*_*ij*_ (10^−4^)	*N*_*e*_
Receiving population																	
Meed	–	–	1.30	7.2	1.55	8.6	1.51	8.4	**2.22**	**12.3**	0.87	4.8	1.30	7.2	1.65	9.2	517.1
Kon	1.09	6.1	–	–	1.58	8.8	1.54	8.6	**1.84**	**10.2**	1.17	6.5	1.30	7.2	1.39	7.7	490.1
Pua	1.57	8.7	0.98	5.4	–	–	1.50	8.3	**1.95**	**10.8**	1.00	5.6	0.83	4.6	1.43	8.0	521.6
Yao	1.34	7.5	1.49	8.3	0.85	4.7	–	–	**1.61**	**9.0**	1.54	8.6	1.42	7.9	1.40	7.8	485.6
Yang	1.66	9.2	1.77	9.8	**2.25**	**12.5**	2.15	12	–	–	1.47	8.2	1.43	8.0	1.58	8.8	490.1
Sa	1.55	8.6	0.97	5.4	1.26	7.0	**1.64**	**9.1**	1.37	7.6	–	–	1.38	7.7	0.95	5.3	485.6
Wa	1.14	6.3	0.93	5.2	0.66	3.7	1.17	6.5	1.17	6.5	**1.39**	**7.7**	–	–	0.84	4.7	481.1
Haeng	1.53	8.5	1.38	7.7	1.33	7.4	1.35	7.5	**1.63**	**9.1**	1.15	6.4	1.18	6.6	–	–	517.1
Average immigration	1.30	7.2	1.30	7.2	0.88	4.9	1.30	7.2	1.30	7.2	1.30	7.2	1.30	7.2	1.30	7.2	–
Average emigration	1.30	7.2	0.88	4.9	1.30	7.2	1.30	7.2	1.93	10.7	1.03	7.2	1.30	7.2	1.30	7.2	–
Immigration-emigration	0	0	0.42	2.3	−0.42	-2.3	0	0	−0.63	-3.5	0	0	0	0	0	0	–

### Spatial pattern of genetic variation

Based on the Pearson correlation coefficients, allelic richness within samples was significantly negatively correlated with stream order of the sampling locations (*P* = 0.015). However, it did not correlate significantly with other landscape attributes, including elevation, distance to the Nan River main stem, number of barriers within tributaries, % forest, % agriculture and % paddy field (*P* > 0.05) (Table 6).

**Table 6:** Pearson correlations between landscape characteristics and allelic richness within *Garra cambodgiensis* population samples. Significant correlations are underlined.

Landscape characteristics	Pearson correlation coefficient	*P*-values (2-tailed)
Elevation	0.467	0.243
Stream order	−0.811	0.015
Distance from the Nan River main stem	−0.523	0.183
Number of barriers within tributaries	0.408	0.315
% Forest	−0.512	0.194
% Agriculture	0.065	0.878
% Paddy field	0.492	0.216

We found a significant correlation between geographic distance (log (geographic distance)) and linearized *F*_ST_ among samples (Mantel *r* = 0.42, one sided *P* = 0.022). Genetic differentiation between populations was moderately explained by contemporary patterns of stream connectivity (*R*^2^ = 0.75 for the STREAMTREE model). However, the difference in elevation between sampling locations did not correlate with genetic distance. Based on the *AIC*c and *w*_*i*_ criteria, two regression models identified log stream distance and pairwise differences in stream orders as important explanatory variables for spatial genetic variation. However, neither of these variables explained much of the spatial genetic variation (*R*^2^ < 0.01) (Table 7).

**Table 7:** Multiple regression on distance matrices (MRM) to test the relationships between landscape variables and linearized pairwise *F*_ST_ among populations of *Garra cambodgiensis*.

Models	*K*	Δ*AIC*c	*w*_*i*_	Cum. *w*_*i*_	Coefficients		MRM	
					LogDIST	STO	*R*^2^	*P*
LogDIST	3	0.0	0.44	0.44	0.0068	–	0.019	0.15
LogDIST + STO	4	1.8	0.18	0.62	0.0068	0.0013	0.033	0.19

**Figure 4:**
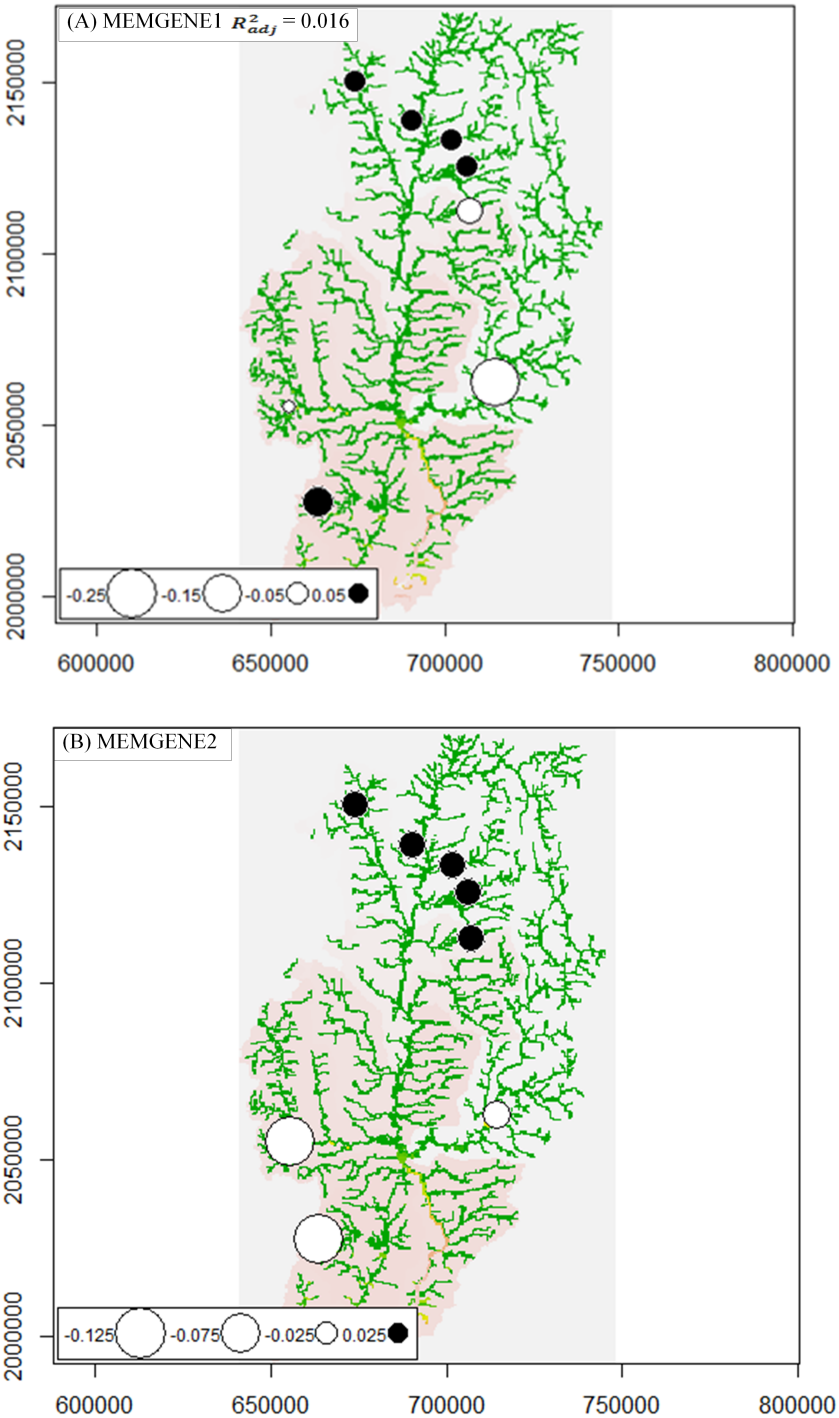
MEMGENE analysis for eight population samples of *Garra cambodgiensis* in the upper Nan River basin. Circles of a similar size and color suggest individuals with similar MEMGENE scores imposed on a map of the upper Nan River basin (large black and large white circle describe opposite extremes on the MEMGENE axes). (A) MEMGENE axis 1 explains 57.8% of the variability and (B) MEMGENE axis 2 explains 42.2%. Both axes indicate spatial genetic differentiation between the northern (upstream) and southern (downstream) sites.

We detected evidence for at least two distinct spatial genetic neighborhoods separated by the north-south division (black and white circles on the map, Fig. 4). The color of the circle indicates the two extremes of the MEMGENE scores. The size of the circle indicates the magnitude. The first two MEMGENE axes described almost all of the variability, with the first MEMGENE axis explaining 57.8% (Fig. 4A) and the second axis explaining 42.2% (Fig. 4B). In total, only a small proportion of genetic variation can be explained by spatial patterns (*R*^2^adj = 0.016), but this was sufficient to identify neighborhoods that correspond to a landscape pattern.

## Discussion

Our results revealed moderate genetic diversity within and among populations of a tropical stream species, *G. cambodgiensis*, in a river system in northern Thailand. Some landscape features were inferred to have an influence on genetic diversity and population structure in this species. Although population genetic structure among these populations exists, we detected high levels of admixture within most populations sampled. We interpret these patterns as driven by stream dynamics under the influence of tropical monsoons and likely impacts of hatchery-assisted supplementary stocking, as we discuss below.

### Genetic diversity

Overall genetic diversity measures found for *G. cambodgiensis* populations from the upper Nan River (average alleles per locus = 10.99 ± 3.00 alleles, allelic richness = 10.12 ± 2.44 alleles, and *H*_*o*_ = 0.59 ± 0.16) were slightly lower than other *Garra* spp., such as Oriental sucking barb (*G. orientalis*) on Hainan Island, China (Su et al., 2013), and the Omani barb (*G. barreimiae)* in the southeastern Arabian Peninsula (Kirchner et al., 2014). It is possible that because most markers (nine of 11) used in our study were developed for other congeneric species, they had fewer polymorphisms in our species. At the set of identical loci also used in our study, a *G. orientalis* population collected from the Wanquan River in Hainan Island (*n* = 23), had much higher microsatellite variation, with allelic diversity ranging from 8 (Gar8) to 25 (Gar9) alleles and expected heterozygosities ranging from 0.72 (Gar8) to 1.0 (Gar6) (Su et al., 2013). At another three loci, PH8A, JQSO, and HOLN, two *G. barreimiae* populations in the southeastern Arabian Peninsula (*n* = 44 from each location) had 7 to 15 alleles per locus (Kirchner et al., 2014). It may also be possible that *G. cambodgiensis* populations in the upper Nan River are much smaller than those of these congeners and therefore, they are subject to a different mutation-drift equilibrium. However, the diversity within populations of widely dispersed *G. cambodgiensis* was higher than that of other tropical freshwater fish species, especially those with more restricted distributions (e.g., Guppy (*Poecilia reticulata*), Barson, Cable & Van Oosterhout, 2009; Northern Purple Spotted Gudgeon (*Mogurnda mogurnda*), Cook et al., 2011).

Stream orders inversely correlated with allelic diversity, although these *G. cambodgienis* populations did not experience recent bottleneck events. This observation reflects the dynamics between species-specific requirements for suitable upstream habitat and other ecological factors facilitating a longer-term maintenance of stable effective population size and high genetic diversity in tributaries of our system. *G. cambodgiensis* is a headwater species, with highest abundance in low-order streams. This species inhabits rocky bottoms with fast-moving water, which are somewhat limited to small and mid-size streams (i.e., lower stream orders). High order streams may be barriers to dispersal. On the other hand, having a reproductive peak during the rainy season with semi-buoyant eggs (Froese & Pauly, 2017) may facilitate extensive admixture between the Nan River main stem and populations in other sub-basins. During the rainy season, breeders often congregate in flooded flat areas (rice paddies) in the watershed downstream of the tributaries (Fig. 1), where adults are typically absent during the dry season (personal observations). To confirm the linkage between life history and location, additional fry samples, identified to species by a molecular technique, may be needed.

Among upper Nan River sites, the Wa sample had the lowest allelic richness (*P* < 0.05, rank test) and slightly lower effective population size based on the sib-ship method (*N*_es_). This sample represents a population inhabiting the downstream segment of the Wa River, in the Nam Wa sub-basin (Fig. 1). Due to the mountainous topology and high elevation, this sub-basin is relatively isolated from other sub-basins. In addition, the Wa River is relatively large (stream orders 5 to 7) and contains stretches of whitewater rapids, potentially serving as physical barriers to fish movements between the main stem Nan River and the Wa River. Population size reduction by habitat fragmentation can lead to low genetic diversity in freshwater fishes (e.g., *G. orientalis*, Yang et al., 2016; Lahontan cutthroat trout (*Oncorhynchus clarkii henshawi*), Neville, Dunham & Peacock, 2006; Brook trout (*Salvelinus fontinalis*), Kanno, Vokoun & Letcher, 2011; Eurasian perch (*Perca fluviatilis*), Yang et al., 2012; River blackfish (*Gadopsis marmoratus*), Lean et al., 2016). For *G. cambodgiensis*, Jaisuk (2018) found that genetic diversity of populations along the entire Wa River were comparable and consistently lower than that of the upper Nan River populations.

### Population genetic structure in the upper Nan River drainage basin

The *G. cambodgiensis* populations in the upper Nan River basin are genetically heterogeneous. The existing population genetic structure can be explained mainly by geographic distance, hierarchical river structure, and life history, although pairwise differences in stream orders between sampling locations may also contribute to this genetic differentiation (although not statistically significant based on the MRM analyses). We detected four genetic clusters according to the river topology: (1) headwater tributaries (Meed/Kon/Pua) and main stem of the Nan River (including Haeng), (2) a middle tributary (Yang), (3) an eastern tributary (Wa), and (4) a western tributary (Sa). Historical migration rates were relatively low among these sub-basins (*N*_*e*_*m* < 0.7). The low genetic differentiation between two headwater streams, Kon and Meed, can be explained by their connecting waterways (only 22.5 km apart). The Yang sample from the second part of the Mae Nam Nan sub-basin was more closely related to the main stem cluster than to the remaining clusters. Wa and Sa represented the cluster most distantly related to the headwater streams/main stem cluster. Isolation by distance and a stream hierarchy structure can explain population genetic structure in stream fish species, especially those with dispersal mediated by current (e.g., Doctor fish (*Gara rufa*), Miandare et al., 2016; Bluehead sucker *(Catostomus discobolus)*, Hopken, Douglas & Douglas, 2012). Similar to our study, Hopken, Douglas & Douglas (2012) discovered a similar effect of a stream hierarchy on the spatial genetic divergence pattern of Bluehead sucker (*Catostomus discobolus*) populations in three large river drainage basins of western North America (i.e., the Colorado River, Snake River, and Bonneville River basins) (significant correlation between river network and genetic distance, STREAMTREE, *R*^2^ = 0.987). For the entire Colorado River basin, there were three evolutionarily significant units (ESUs) of *C. discobolus* populations divided by segments of the river.

Although the isolation-by-distance pattern of genetic differentiation can be observed in multiple fish species, the geographic distance at which populations diverge varies with the species’ dispersal ability and the degree of restrictions of river flow. For *G. cambodgiensis* in our study, flooding can facilitate genetic homogeneity for locations along the main stem (more than 100 km apart), but the landscape disconnectivity among sub-basins restricts gene flow between sites in adjacent sub-basins (i.e., the upper part of Mae Nam Nan basin vs. the second part of Mae Nam Nan sub-basins). In Brook trout (*Salvelinus fontinalis*), Castric, Bonny & Bernatchez (2001) found different patterns of spatial genetic variation in population samples between two neighboring rivers in eastern North America. The population divergence pattern in the Penobscot River, Maine could be explained by the isolation by distance, whereas the pattern in the Saint John River, New Brunswick could not. Similarly, Crookes & Shaw (2016) could detect a positive correlation between genetic structuring (*F*_ST_) and distance (20–25 km) in populations of common roach (*Rutilus rutilus*) in only one of the two rivers studied, the Stour River, in southeast England.

A surprising finding that conformed to neither the isolation-by-distance pattern nor to the upper Nan River hierarchy structure was high genetic similarity between the Sa and Yao samples (*F*_ST_ = 0.003), located more than 190 km apart in a different sub-basin. The model-based clustering analyses suggested that an upstream site, the Yao River (in the eastern Nam Yao sub-basin), contained genetic material from at least two sources, the main stem cluster and the western tributary cluster (Nam Sa). A similar pattern has been reported by Beneteau, Mandrak & Heath (2009), who found that population samples of Greenside darter (*Etheostoma blennioides*) from two separate drainages, the Sydenham and Thames rivers, Ontario, Canada, were genetically similar (mean pairwise *F*_ST_ = 0.016). A possible explanation for this weak divergence was an on-going gene flow through Lake St. Clair, an adjacent water body connecting the two rivers. Similarly, Davis, Wieman & Berendzen (2015) reported lack of genetic differentiation among Rainbow darter (*Etheostoma caeruleum*) samples across four tributaries of the upper Mississippi River (only 1.05% of the genetic variation was attributed to among-drainage variation, even at sites located 60–120 km apart). The authors hypothesized that the northward population expansion of this species occurred recently, after the retreat of the last glaciation event (15,000 years ago). This historical process overwhelmed more recent genetic changes due to life histories (e.g., strict habitat requirements) and a contemporary river structure network (STREMTREE analysis, *R* ^2^ = 0.578). For *G. cambodgiensis* in the Nam Yao-1 and Nam Sa sub-basins, the two sub-basins lie on the same side of a ridge (the east Phi Pun Nam range), and the headwater streams may have been connected in the past. In addition, the two sub-basins are adjacent to the sub-basin of another large tributary of the Chao Phraya River, the Yom River, and partially connected through tributary streams of the Yom River, especially during flooding. For example, the Huai Mae Tha Stream, a tributary stream of the Yom River, flows directly into Sa River. It is possible that *G. cambodginensis* populations in Sa River share some ancestry with a population in the Yom River. Genetic analysis of additional individuals from the Yom River could be helpful.

### Admixture and gene flow among populations

Although most genetic clusters defined by the Bayesian clustering analysis correspond to the river topology and the sub-basin division, the TESS bar plots based on membership coefficients of individuals (Fig. 3) suggested extensive admixture in most locations. This admixture can be a result of occasional mixing of breeders during the flood season and of supplementary stocking of fry, which started in 2009 (Table 8). The natural admixture could happen during the rainy season, which typically coincides with the reproductive season of several fish species, including *G. cambodgiensis* (a peak in June-July, Paugy, 2002). During this period, most of the downstream segments of rivers in sub-basins would be occasionally flooded, allowing breeders to congregate in suitable breeding habitats (rice paddies) from neighboring sub-basins (Fig. 1). As the floods recede, fish fry usually drift to suitable nursery/feeding habitats. The consequence of genetic exchange via flooding is evident by the presence of admixed individuals containing genetic material from the main stem cluster in almost every site in the upper Nan river sub-basins. Moreover, the most downstream site, the Haeng sample, contained genetic material from all genetic clusters, including those of adjacent sub-basins. This admixture may also have resulted in high numbers of heterozygote deficiency (Wahlund effect).

**Table 8:** Records of supplementary stocking activities within the upper Nan River drainage basin between 2009 and 2017.

Years	Source of brooders	Released site	Number of individual fry released
2009	Wa River	Wa River	200,000
2011	Wa River	Sa River	200,000
2013	Yang River	Kon River	200,000
	Yang River	Yang River	100,000
2014	Kon and Yang Rivers	Kon River	200,000
	Wa River	Wa River	200,000
2015	Kon and Meed Rivers	Kon River	200,000
	Kon and Meed Rivers	Meed River	10,000
2016	Kon and Meed Rivers	Kon River	150,000
	Kon and Yang Rivers	Kon River	50,000
	Wa River	Wa River	200,000
2017	Kon River	Kon River	200,000
	Wa River	Wa River	200,000
	Yang River	Yang River	200,000

This pattern of admixture is rather common in tropical monsoonal rivers and streams, especially for smaller-bodied fish species (e.g., Oriental sucking barb (*Garra orientalis*), Yang et al., 2016; Guppy (*Poecilia reticulata),* Barson, Cable & Van Oosterhout, 2009; Purple Spotted Gudgeon (*Mogurnda adspersa*), Hughes et al., 2012; Three-spined stickleback (*Gasterosteus aculeatus*), Hanson et al., 2016). For example, Yang et al. (2016) reported that two lowland populations of *G orientalis*, in the Pearl River, China, contained highly admixed individuals compared to other sampling sites. In *P. reticulata* populations from Trinidad and Tobago, Barson, Cable & Van Oosterhout (2009) hypothesized that the fast-flowing, turbid waters during the wet season forced a strong downstream migration and resulted in admixed populations.

Historical migration rates among sites in this study are estimated to be relatively low (*N*_*e*_*m* < 0.7), and there is evidence of slight genetic impacts of supplementary stocking of fry in some locations. The supplementary stocking program was initiated in 2009 and released approximately 200,000 individuals per site per year (personal observations, Table 8). Broodstock were collected from the wild and used for only one generation. In most cases, the breeder source and release site were the same (e.g., Wa River). However, for the cross sub-basin movements, the majority of the movements were between the Yang River from the second part of the Mae Nam Nan sub-basin to the Kon/Meed Rivers in the upper part of the Mae Nam Nan sub-basin (in 2013, 2014 and 2016). It is therefore not surprising to observe a genetic contribution from the Yang genetic cluster in the headwater streams and the main stem locations. Compared to other sites, relatively higher migration rates apparent between the Yang River and other sites within the main stem cluster suggested such movements (*N*_*e*_*m* ∼0.178–0.636, Tables 5 and 8). The past stocking, however, has not altered existing population genetic structure (i.e., Yang is still genetically distinct from the upstream sites).

### Management implications—possible metapopulation dynamics in *G. cambodgiensis* populations in upper Nan River

One of the key long-term conservation goals is to protect the evolutionary potential of populations (Allendorf & Luikart, 2007). Our results provide insights potentially useful for achieving this goal. For within-population genetic diversity, we have not detected recent bottleneck events or low effective population size in the populations studied. However, extreme fishing pressure, especially in the spawning seasons, should be closely monitored and possibly regulated if there is evidence of overfishing. For population divergence, based on 11 polymorphic microsatellite loci, the genetic analyses revealed at least four distinct genetic clusters of *G. cambodgiensis* populations that corresponded roughly to the distinct sub-basins of the upper Nan River drainage system, including the second part of the Mae Nam Nan (Yang), Nam Sa, Nam Wa (Wa) and the remaining portion of the upper Nan River/main stem. This spatial-genetic divergence pattern can be used as a guide for identifying conservation and fishery management units in the upper Nan River drainage basin. Any supplementary stocking using broodstock from a different sub-basin should be avoided.

The high degree of admixture among genetically distinct populations highlights the importance of natural flooding patterns, allowing for admixture, and may reflect the genetic impacts of supplemental stocking. It is possible that this fish species has metapopulation dynamics, with the main stem of the upper Nan River and annual flooding facilitating connectivity among demes. Due to clear sub-division among some sub-basins, future supplemental stocking programs should be planned more carefully (Miller & Kapuscinski, 2003). In addition, to refine appropriate conservation schemes for this area, we need to explore potential genetic subdivision in some complex sub-basins (e.g., Wa River) with man-made and natural barriers.

## Conclusions

This work is among a few studies providing insights into population genetics of tropical stream fishes in Southeast Asia, a hotspot for freshwater biodiversity. Our work highlights the importance of the historical and contemporary stream network as well as monsoonal flooding patterns on genetic connectivity among the populations of different sub-basins. In addition, the spatial genetic variation of *G. cambodgiensis* populations in the upper Nan River, Thailand, suggests interactions between habitat isolation (due to specialized habitat requirements) and the flood-mediated dispersal pattern. The high and consistent level of admixture in most populations suggests possible metapopulation dynamics for this species. The genetic analysis also suggests slight genetic impacts of a recent hatchery-assisted stocking program in the area and cautions against extensive reliance on use of broodstock from a different sub-basin.

## Supplemental Information

10.7717/peerj.4487/supp-1 Supplemental Information 1 Multilocus microsatellite genotypes of *G. cambodgiensis* collected from upper Nan River, Thailand, during November to December 2016

10.7717/peerj.4487/supp-2 Table S1 *F*_*ST*_ values estimated for each pair of populations using the ENA, excluding null alleles correction method described in Chapuis & Estoup (2007)

10.7717/peerj.4487/supp-3 Table S2 Allelic variability at 11 microsatellite loci of *Garra cambodgiensis* populations in the upper Nan River, Thailand The indices included the sample size (*N*), number of alleles per locus (*A*), effective number of alleles (*A*_*e*_), allelic richness (*A*_*r*_), observed heterozygosity (*H*_*o*_), expected heterozygosity (*H*_*e*_), estimated null allele frequencies and fixation index (*F*_*is*_) values and probability of significant deviation from Hardy-Weinberg equilibrium (*P*) are given for each population and locus. Values underlined indicate statistical significance, *P* < 0.00057, after Bonferroni correction = 0.05/88.

## Additional Information and Declarations

### Data Availability

The following information was supplied regarding data availability: The multilocus microsatellite genotypes of *G. cambodgiensis* collected from the upper Nan River, Thailand, during January to February 2015 are provided in a Supplemental File.
