# Supplementary material for: Effects of landscape features on population genetic variation of a tropical stream fish, Stone lapping minnow, Garra cambodgiensis, in the upper Nan River drainage basin, northern Thailand
Source: PeerJ. 2018 Mar 7;6:e4487. doi: 10.7717/peerj.4487 (PMC5845392; doi:10.7717/peerj.4487)
Supplement: Table S1 [file peerj-06-4487-s002.docx]

|  | Meed | Kon | Pua | Yao | Yang | Sa | Wa | Haeng |
| --- | --- | --- | --- | --- | --- | --- | --- | --- |
| Meed | - |  |  |  |  |  |  |  |
| Kon | 0.003 | - |  |  |  |  |  |  |
| Pua | 0.020 | 0.010 | - |  |  |  |  |  |
| Yao | 0.015 | 0.013 | 0.015 | - |  |  |  |  |
| Yang | 0.021 | 0.022 | 0.021 | 0.021 | - |  |  |  |
| Sa | 0.033 | 0.028 | 0.021 | 0.005 | 0.030 | - |  |  |
| Wa | 0.047 | 0.043 | 0.038 | 0.043 | 0.052 | 0.045 | - |  |
| Haeng | 0.020 | 0.016 | 0.014 | 0.018 | 0.020 | 0.024 | 0.054 | - |
