# Supplementary material for: Effects of landscape features on population genetic variation of a tropical stream fish, Stone lapping minnow, Garra cambodgiensis, in the upper Nan River drainage basin, northern Thailand
Source: PeerJ. 2018 Mar 7;6:e4487. doi: 10.7717/peerj.4487 (PMC5845392; doi:10.7717/peerj.4487)
Supplement: Table S2 — The indices included the sample size (N), number of alleles per locus (A), effective number of alleles (Ae), allelic richness (Ar), observed heterozygosity (Ho), expected heterozygosity (He), estimated null allele frequencies and fixation index (Fis) values and probability of significant deviation from Hardy-Weinberg equilibrium (P) are given for each population and locus. Values underlined indicate statistical significance, P < 0.00057, after Bonferroni correction = 0.05/88. [file peerj-06-4487-s003.docx]

| locations | Locus | *N* | *A* | *A*_e_ | *A*_r_ | *H*_o_ | *H*_e_ | *F*_is_ | Null allele frequency |
| --- | --- | --- | --- | --- | --- | --- | --- | --- | --- |
| Meed | Gar3 | 41 | 10 | 6.18 | 9.65 | 0.61 | 0.84 | 0.27 | 0.13 |
|  | Gar6 | 43 | 13 | 6.64 | 12.10 | 0.70 | 0.85 | 0.18 | 0.09 |
|  | Gar8 | 46 | 5 | 3.89 | 4.88 | 0.46 | 0.74 | 0.39 | 0.17 |
|  | Gar9 | 42 | 10 | 4.24 | 9.31 | 0.36 | 0.76 | 0.53 | 0.23 |
|  | Gar13 | 42 | 8 | 4.27 | 7.83 | 0.57 | 0.77 | 0.25 | 0.10 |
|  | GC187 | 44 | 11 | 7.55 | 10.62 | 0.55 | 0.87 | 0.37 | 0.18 |
|  | GC203 | 44 | 12 | 8.59 | 11.35 | 0.73 | 0.88 | 0.18 | 0.10 |
|  | HOLN | 45 | 13 | 8.22 | 12.07 | 0.44 | 0.88 | 0.49 | 0.23 |
|  | JQSO | 46 | 10 | 6.98 | 9.85 | 0.61 | 0.86 | 0.29 | 0.15 |
|  | Sa197 | 42 | 15 | 9.31 | 14.14 | 0.33 | 0.89 | 0.63 | 0.29 |
|  | PH8A | 43 | 13 | 3.94 | 12.00 | 0.30 | 0.75 | 0.60 | 0.26 |
|  | Ave. | 43.46±1.62 | 10.91±2.64 | 6.35±1.90 | 10.35±2.38 | 0.51±0.14 | 0.83±0.06 | 0.38±0.15 | 0.18±0.07 |
| Kon | Gar3 | 45 | 12 | 6.23 | 10.49 | 0.78 | 0.84 | 0.07 | 0.04 |
|  | Gar6 | 46 | 17 | 7.50 | 14.65 | 0.65 | 0.87 | 0.25 | 0.12 |
|  | Gar8 | 46 | 5 | 3.94 | 4.88 | 0.59 | 0.75 | 0.21 | 0.09 |
|  | Gar9 | 38 | 13 | 3.06 | 11.60 | 0.37 | 0.67 | 0.45 | 0.19 |
|  | Gar13 | 41 | 11 | 5.20 | 9.85 | 0.56 | 0.81 | 0.31 | 0.13 |
|  | GC187 | 45 | 9 | 6.05 | 8.95 | 0.60 | 0.84 | 0.28 | 0.12 |
|  | GC203 | 46 | 11 | 6.38 | 10.47 | 0.67 | 0.84 | 0.20 | 0.10 |
|  | HOLN | 43 | 12 | 8.25 | 11.55 | 0.44 | 0.88 | 0.50 | 0.23 |
|  | JQSO | 46 | 12 | 6.22 | 11.03 | 0.74 | 0.84 | 0.12 | 0.07 |
|  | Sa197 | 43 | 11 | 5.12 | 10.08 | 0.26 | 0.80 | 0.68 | 0.30 |
|  | PH8A | 45 | 15 | 3.55 | 13.13 | 0.51 | 0.72 | 0.29 | 0.13 |
|  | Ave. | 44±2.45 | 11.64±2.93 | 5.59±1.54 | 10.61±2.36 | 0.56±0.15 | 0.81±0.06 | 0.31±0.17 | 0.14±0.07 |
| Pua | Gar3 | 46 | 11 | 7.60 | 10.17 | 0.74 | 0.87 | 0.15 | 0.06 |
|  | Gar6 | 46 | 16 | 7.50 | 14.48 | 0.76 | 0.87 | 0.12 | 0.06 |
|  | Gar8 | 46 | 9 | 4.02 | 7.49 | 0.61 | 0.75 | 0.19 | 0.09 |
|  | Gar9 | 43 | 10 | 2.25 | 8.79 | 0.33 | 0.56 | 0.41 | 0.16 |
|  | Gar13 | 46 | 13 | 6.74 | 12.11 | 0.72 | 0.85 | 0.16 | 0.07 |
|  | GC187 | 45 | 11 | 7.17 | 10.82 | 0.56 | 0.86 | 0.35 | 0.16 |
|  | GC203 | 46 | 12 | 6.11 | 11.47 | 0.83 | 0.84 | 0.01 | 0.02 |
|  | HOLN | 46 | 13 | 9.14 | 11.84 | 0.74 | 0.89 | 0.17 | 0.09 |
|  | JQSO | 46 | 11 | 7.43 | 10.15 | 0.87 | 0.87 | -0.01 | 0.02 |
|  | Sa197 | 43 | 14 | 8.83 | 13.35 | 0.51 | 0.89 | 0.42 | 0.20 |
|  | PH8A | 45 | 13 | 1.83 | 10.89 | 0.27 | 0.46 | 0.41 | 0.14 |
|  | Ave. | 45.27±1.14 | 12.09±1.88 | 6.24±2.36 | 11.05±1.87 | 0.63±0.19 | 0.79±0.14 | 0.22±0.15 | 0.10±0.06 |
| Yao | Gar3 | 46 | 10 | 6.61 | 9.53 | 0.72 | 0.85 | 0.16 | 0.06 |
|  | Gar6 | 46 | 15 | 5.28 | 12.71 | 0.67 | 0.81 | 0.17 | 0.07 |
|  | Gar8 | 46 | 6 | 4.02 | 5.64 | 0.61 | 0.75 | 0.19 | 0.07 |
|  | Gar9 | 43 | 11 | 3.96 | 10.18 | 0.49 | 0.75 | 0.35 | 0.15 |
|  | Gar13 | 46 | 10 | 4.53 | 9.51 | 0.61 | 0.78 | 0.22 | 0.11 |
|  | GC187 | 46 | 9 | 5.85 | 8.52 | 0.67 | 0.83 | 0.19 | 0.09 |
|  | GC203 | 46 | 13 | 7.78 | 12.67 | 0.83 | 0.87 | 0.05 | 0.03 |
|  | HOLN | 44 | 12 | 6.96 | 11.58 | 0.64 | 0.86 | 0.26 | 0.13 |
|  | JQSO | 46 | 11 | 8.46 | 10.64 | 0.85 | 0.88 | 0.04 | 0.02 |
|  | Sa197 | 45 | 14 | 7.60 | 12.75 | 0.44 | 0.87 | 0.49 | 0.23 |
|  | PH8A | 44 | 14 | 3.83 | 12.45 | 0.59 | 0.74 | 0.20 | 0.10 |
|  | Ave. | 45.27±1.05 | 11.36±2.50 | 5.90±1.61 | 10.56±2.11 | 0.65±0.12 | 0.82±0.05 | 0.21±0.12 | 0.10±0.06 |
| **Yang** | Gar3 | 99 | 14 | 6.49 | 11.39 | 0.71 | 0.85 | 0.16 | 0.07 |
|  | Gar6 | 98 | 23 | 8.00 | 16.88 | 0.62 | 0.88 | 0.29 | 0.14 |
|  | Gar8 | 100 | 8 | 3.94 | 5.83 | 0.57 | 0.75 | 0.24 | 0.11 |
|  | Gar9 | 81 | 14 | 3.74 | 10.89 | 0.31 | 0.73 | 0.58 | 0.25 |
|  | Gar13 | 99 | 13 | 5.92 | 9.88 | 0.58 | 0.83 | 0.31 | 0.14 |
|  | GC187 | 93 | 11 | 6.61 | 10.56 | 0.54 | 0.85 | 0.37 | 0.17 |
|  | GC203 | 99 | 12 | 9.71 | 11.44 | 0.66 | 0.90 | 0.27 | 0.13 |
|  | HOLN | 96 | 15 | 10.05 | 13.18 | 0.64 | 0.90 | 0.29 | 0.14 |
|  | JQSO | 100 | 12 | 6.31 | 10.10 | 0.75 | 0.84 | 0.11 | 0.05 |
|  | Sa197 | 91 | 13 | 3.32 | 10.91 | 0.24 | 0.70 | 0.65 | 0.28 |
|  | PH8A | 96 | 12 | 2.36 | 8.37 | 0.48 | 0.58 | 0.17 | 0.10 |
|  | Ave. | 95.64±5.4 | 13.36±3.52 | 6.04±2.42 | 10.86±2.62 | 0.55±0.15 | 0.80±0.10 | 0.31±0.16 | 0.14±0.07 |
| Sa | Gar3 | 40 | 10 | 5.16 | 9.87 | 0.73 | 0.81 | 0.10 | 0.03 |
|  | Gar6 | 41 | 10 | 4.82 | 9.42 | 0.81 | 0.79 | -0.02 | 0.00 |
|  | Gar8 | 41 | 6 | 3.28 | 5.90 | 0.59 | 0.70 | 0.16 | 0.04 |
|  | Gar9 | 36 | 8 | 2.00 | 7.62 | 0.25 | 0.50 | 0.50 | 0.19 |
|  | Gar13 | 38 | 10 | 5.80 | 9.86 | 0.55 | 0.83 | 0.33 | 0.15 |
|  | GC187 | 41 | 9 | 5.81 | 8.66 | 0.54 | 0.83 | 0.35 | 0.16 |
|  | GC203 | 41 | 12 | 8.36 | 11.65 | 0.76 | 0.88 | 0.14 | 0.08 |
|  | HOLN | 39 | 12 | 7.47 | 11.60 | 0.69 | 0.87 | 0.20 | 0.09 |
|  | JQSO | 41 | 11 | 8.53 | 10.66 | 0.81 | 0.88 | 0.09 | 0.04 |
|  | Sa197 | 35 | 10 | 6.46 | 9.71 | 0.57 | 0.85 | 0.32 | 0.15 |
|  | PH8A | 40 | 10 | 3.67 | 9.36 | 0.65 | 0.73 | 0.11 | 0.06 |
|  | Ave. | 39.36±2.06 | 9.82±1.64 | 5.58±1.99 | 9.48±1.59 | 0.63±0.15 | 0.79±0.11 | 0.21±0.14 | 0.09±0.06 |
| Wa | Gar3 | 30 | 8 | 4.95 | 8.00 | 0.63 | 0.80 | 0.21 | 0.09 |
|  | Gar6 | 30 | 8 | 3.30 | 8.00 | 0.63 | 0.70 | 0.09 | 0.05 |
|  | Gar8 | 30 | 4 | 2.58 | 4.00 | 0.73 | 0.61 | -0.20 | 0.00 |
|  | Gar9 | 30 | 8 | 2.81 | 8.00 | 0.63 | 0.64 | 0.02 | 0.03 |
|  | Gar13 | 30 | 6 | 2.54 | 6.00 | 0.57 | 0.61 | 0.07 | 0.04 |
|  | GC187 | 30 | 8 | 4.60 | 8.00 | 0.77 | 0.78 | 0.02 | 0.01 |
|  | GC203 | 30 | 11 | 7.35 | 11.00 | 0.77 | 0.86 | 0.11 | 0.06 |
|  | HOLN | 30 | 10 | 6.52 | 10.00 | 0.57 | 0.85 | 0.33 | 0.15 |
|  | JQSO | 30 | 8 | 5.94 | 8.00 | 0.70 | 0.83 | 0.16 | 0.07 |
|  | Sa197 | 30 | 8 | 4.80 | 8.00 | 0.53 | 0.79 | 0.33 | 0.13 |
|  | PH8A | 30 | 5 | 2.07 | 5.00 | 0.30 | 0.52 | 0.42 | 0.16 |
|  | Ave. | 30±0 | 8.00±1.92 | 4.31±1.70 | 7.64±1.92 | 0.62±0.13 | 0.73±0.11 | 0.14±0.17 | 0.07±0.05 |
| Haeng | Gar3 | 40 | 12 | 8.51 | 11.47 | 0.70 | 0.88 | 0.21 | 0.10 |
|  | Gar6 | 42 | 15 | 6.49 | 13.51 | 0.50 | 0.85 | 0.41 | 0.19 |
|  | Gar8 | 42 | 7 | 3.37 | 6.35 | 0.48 | 0.70 | 0.32 | 0.13 |
|  | Gar9 | 38 | 6 | 2.07 | 5.75 | 0.42 | 0.52 | 0.19 | 0.08 |
|  | Gar13 | 34 | 10 | 6.35 | 9.75 | 0.62 | 0.84 | 0.27 | 0.12 |
|  | GC187 | 42 | 10 | 7.38 | 9.91 | 0.57 | 0.87 | 0.34 | 0.17 |
|  | GC203 | 40 | 11 | 6.03 | 10.48 | 0.48 | 0.83 | 0.43 | 0.20 |
|  | HOLN | 41 | 15 | 10.22 | 14.29 | 0.63 | 0.90 | 0.30 | 0.14 |
|  | JQSO | 42 | 11 | 7.84 | 10.40 | 0.83 | 0.87 | 0.05 | 0.02 |
|  | Sa197 | 38 | 14 | 8.62 | 13.34 | 0.21 | 0.88 | 0.76 | 0.36 |
|  | PH8A | 41 | 11 | 3.41 | 9.65 | 0.46 | 0.71 | 0.34 | 0.14 |
|  | Ave. | 40±2.37 | 11.09±2.78 | 6.39±2.41 | 10.45±2.59 | 0.54±0.16 | 0.81±0.11 | 0.33±0.17 | 0.15±0.08 |
| All samples | Ave. | 47.88±18.82 | 10.99±3.00 | 5.80±2.12 | 10.12±2.44 | 0.59±0.16 | 0.80±0.10 | 0.26±0.17 | 0.12±0.07 |
| Each locus | Gar3 | 48.38±19.74 | 10.88±1.69 | 6.47±1.10 | 10.07±1.04 | 0.70±0.05 | 0.84±0.03 | 0.17±0.06 | 0.07±0.03 |
|  | Gar6 | 49.00±19.16 | 14.63±4.27 | 6.19±1.50 | 12.72±2.70 | 0.67±0.09 | 0.83±0.06 | 0.19±0.12 | 0.09±0.05 |
|  | Gar8 | 49.63±19.71 | 6.25±1.56 | 3.63±0.48 | 5.62±0.99 | 0.58±0.08 | 0.72±0.05 | 0.24±0.07 | 0.09±0.05 |
|  | Gar9 | 43.88±14.61 | 10.00±2.50 | 3.02±0.83 | 9.02±1.78 | 0.40±0.11 | 0.64±0.10 | 0.38±0.18 | 0.16±0.07 |
|  | Gar13 | 47.00±20.33 | 10.13±2.20 | 5.17±1.27 | 9.35±1.66 | 0.60±0.05 | 0.79±0.07 | 0.24±0.08 | 0.11±0.03 |
|  | GC187 | 48.25±17.58 | 9.75±1.09 | 6.38±0.93 | 9.51±1.03 | 0.60±0.08 | 0.84±0.03 | 0.28±0.11 | 0.13±0.05 |
|  | GC203 | 49.00±19.55 | 11.75±0.66 | 7.54±1.24 | 11.32±0.66 | 0.72±0.11 | 0.86±0.02 | 0.17±0.12 | 0.09±0.05 |
|  | HOLN | 48.00±18.75 | 12.75±1.56 | 8.35±1.28 | 12.01±1.18 | 0.60±0.10 | 0.88±0.02 | 0.32±0.11 | 0.15±0.05 |
|  | JQSO | 49.63±19.71 | 10.75±1.20 | 7.21±0.95 | 10.10±0.87 | 0.77±0.08 | 0.86±0.02 | 0.11±0.09 | 0.05±0.04 |
|  | Sa197 | 45.88±18.90 | 12.38±2.45 | 6.76±2.18 | 11.54±2.18 | 0.39±0.14 | 0.83±0.07 | 0.54±0.17 | 0.24±0.07 |
|  | PH8A | 48.00±18.71 | 11.63±2.91 | 3.08±0.80 | 10.11±2.47 | 0.45±0.13 | 0.65±0.11 | 0.32±0.15 | 0.14±0.06 |
